# Integration of VectorCam, an AI-Enabled Mosquito Surveillance Tool, Into Uganda’s National Malaria Elimination Program: Protocol for a Mixed Methods Hybrid Type III Implementation Study

**DOI:** 10.2196/99717

**Published:** 2026-09-10

**Authors:** Mei-Li Hey, Marina Rincon Torroella, Diwakar Mohan, Sunny Patel, Winnifred Kansiime, Catherine Maiteki-Sebuguzi, Aryaman Shodhan, Youseph Yazdi, Peter Waiswa, Soumyadipta Acharya

**Affiliations:** 1 Department of Environmental Health and Engineering Bloomberg School of Public Health Johns Hopkins University Baltimore, MD United States; 2 Department of Biomedical Engineering Whiting School of Engineering Johns Hopkins University Baltimore, MD United States; 3 Vector Control Innovations, Inc Ellicott City, MD United States; 4 Department of International Health Bloomberg School of Public Health Johns Hopkins University Baltimore, MD United States; 5 Department of Disease Control and Environmental Health School of Public Health Makerere University Kampala, Central Region Uganda; 6 Uganda National Malaria Elimination Division Ministry of Health Kampala, Central Region Uganda; 7 Department of Health Policy, Planning, and Management School of Public Health Makerere University Kampala, Central Region Uganda

**Keywords:** vector surveillance, malaria, digital technology, implementation research, AI

## Abstract

**Background:**

Malaria remains one of the most significant global health threats, and its control depends on timely, accurate entomological surveillance. In Uganda, routine vector surveillance relies heavily on microscope-based mosquito identification by a limited number of trained entomologists, which can delay reporting. VectorCam is an AI-enabled digital tool to support real-time mosquito identification and can be operated by community health workers, supporting rapid data reporting. After co-designing the implementation package using a human-centered design approach with Uganda’s Ministry of Health, we are implementing a surveillance strategy that incorporates VectorCam into routine work.

**Objective:**

This study aims to evaluate acceptability, fidelity, feasibility, cost, and preliminary effectiveness of VectorCam, the intervention, along with supporting implementation strategies as it is integrated into Uganda’s national malaria control program.

**Methods:**

This mixed methods hybrid type III implementation-effectiveness study uses district-level restricted randomization to assign 12 districts to four sequential rollout waves following a 2-district pilot phase. The implementation package will be introduced over a one-year period across malaria-endemic districts in Northern and Eastern Uganda. Implementation outcomes, including acceptability, feasibility, fidelity, and cost, will be assessed using qualitative interviews, system usage logs, field observations, and program documentation. Effectiveness outcomes include technical performance of mosquito species identification, time efficiency, and operational use of surveillance data for decision-making. Accuracy will be evaluated through comparison with molecular identification methods, alongside additional classification performance metrics. Data will be analyzed using a mixed methods approach, with triangulation of qualitative and quantitative data and longitudinal analyses across districts and rollout waves. An adaptive learning approach will guide iterative refinement of the implementation package.

**Results:**

Fourteen districts were selected by the Ministry of Health in September 2025 for VectorCam introduction. A pilot phase conducted between December 2025 and January 2026 in two districts was used to refine implementation strategies and research tools through stakeholder workshops. Ethical approval was obtained from Johns Hopkins in January 2026 and Ugandan ethics committees in September 2025. Implementation began in February 2026 and will continue through March 2027 with concurrent data collection and analysis throughout the implementation timeline.

**Conclusions:**

This study will evaluate a task-shifting approach to entomological surveillance by integrating an AI-enabled tool into routine workflows of community health workers through a co-designed implementation package. By examining how the VectorCam intervention and supporting implementation strategies function together within real-world health system contexts, this study will generate evidence on the sustainability of digitally enabled surveillance. Findings will provide insight into how task shifting and digital tools can expand surveillance capacity, improve timeliness of data reporting, and strengthen the use of entomological data for decision-making. These results will inform national scale-up efforts in Uganda and offer a model for other malaria-endemic settings seeking to modernize vector surveillance.

## Introduction

### Background

Malaria is a major public health challenge globally, with an estimated 263 million cases and nearly 600,000 deaths recorded in 2023 [1]. Africa is disproportionately affected, as 94% of all malaria cases and 95% of deaths occur on this continent [2]. In Uganda, malaria is endemic in nearly all of the 146 districts and accounts for approximately 30% to 50% of outpatient visits and hospital admissions [3,4]. Despite progress in malaria control over the past 2 decades, recent years have seen a plateau in health gains, highlighting the need for more adaptive, data driven, and locally tailored strategies to work toward elimination [1,5].

A cornerstone of effective malaria control and elimination is timely, accurate entomological surveillance [6,7]. Vector surveillance provides essential data on mosquito species composition, abundance, behavior, and insecticide resistance, all of which are critical for informing targeted interventions such as long-lasting insecticide-treated nets, indoor residual spraying, and larval source management [8-10]. For example, the most common malaria vectors in Uganda are *Anopheles gambiae s.l.* and *Anopheles funestus s.l.* [4]. Previous studies found that certain interventions are more effective in targeting these mosquito groups than others, a fact that underscores the importance of improving the efficiency of surveillance data collection and usage when choosing the correct intervention [11].

However, Uganda and similar locales have a major challenge: the existing vector surveillance systems are limited by their reliance on manual, microscope-based identification of mosquitoes by trained entomologists [12,13]. Uganda’s Ministry of Health (MOH) conducts routine monthly mosquito surveillance through district vector control officers (VCOs). The method currently used is a manual process in which mosquitoes are collected, primarily through pyrethrum spray catches (PSCs), human-landing catches (HLCs), or Centers for Disease Control light trap catches (CDC-LTC), and then identified individually under a microscope to determine the genus and species [14-16]. The data are recorded, aggregated, and manually uploaded to centralized health informatics systems such as District Health Information System 2 (DHIS2). The Vector Control Division then uses these data for decision-making and mosquito population mapping [17].

The current vector surveillance methods are labor- and time-intensive and require special expertise. Therefore, generating actionable insights at the pace needed for effective malaria control is challenging [16]. These limitations delay decision-making and reduce the ability to respond rapidly to changes in mosquito populations and malaria transmission dynamics. To address these surveillance gaps, VectorCam was developed at the Johns Hopkins University, Center for Bioengineering Innovation and Design as a low-cost, AI-enabled tool that allows village health teams (VHTs) and other community health workers to perform mosquito identification tasks without prior entomological training [18].

### VectorCam, a Field Tool for Rapid Morphological Identification of Mosquitoes

AI-enabled tools are quickly gaining recognition as some of the most effective ways to improve data quality and dissemination [19-23]. The VectorCam platform uses a novel combination of a low-cost, 3D-printed device, smartphone-based microscopy, AI, and a mobile app to enable real-time mosquito identification. The devices use a convolutional neural network (CNN), running locally on a low-cost smartphone to photographically identify species, sex, and abdominal status of each mosquito specimen [18]. Then, the data are uploaded to a central server, which generates reports that are automatically sent to malaria control teams. VectorCam is designed to work in offline and low-bandwidth environments and integrates easily into existing workflows. A feasibility study by Couret et al [24] applied a similar CNN to images of mosquitoes for classification of sex, genus, species, and strains and found accuracy rates of up to 98%. This type of technology has implications in low-resource settings where AI-assisted technologies could close critical capacity gaps, such as when trained entomologists and surveillance infrastructure are limited.

In 2023-2024, a randomized controlled trial (RCT) conducted in 2 high malaria-burden districts of Uganda (Mayuge and Adjumani) demonstrated VectorCam’s efficacy and feasibility [25]. During the RCT’s study period, VectorCam generated predictions for primary malaria vectors including *Anopheles gambiae s.l.* (AG) and *Anopheles funestus s.l.* (AF), a heterogeneous grouping labeled Anopheles Other (AO) representing locally relevant secondary vectors, Culicine genera including Culex spp. (CU), Mansonia spp. (MAN), Aedes spp. (AED), and a nonmosquito class to screen out other arthropods commonly captured during PSCs and CDC-LTCs. Across all taxonomic categories, VectorCam achieved an overall identification accuracy of 93% when compared with the gold-standard entomological identification methods [25]. The VectorCam technology also scored above the 90th percentile on the System Usability Scale, indicating that perceived usability is excellent [25]. Additionally, over 77,000 mosquitoes were identified by 24 VHTs over the 12-month study, with an average processing time of 17 seconds per specimen. This rate is an approximately two-fold improvement over the routine, microscope-based method of mosquito identification, which averages 34.5 seconds per specimen. Additionally, the VectorCam arm of the study accomplished a 98% data completion rate (29,785 entries completed out of 30,360 entries possible), compared to 45% (13,667/30,360) in the paper-based, routine surveillance control arm. These percentages reflect the number of mosquito data entries at day 14 (ie, the standard lag time between data collection and submission in Uganda’s routine surveillance workflow) over an 11-month period and across 240 households (120 per arm). By addressing 2 persistent bottlenecks of limited entomological expertise and the slow pace of manual data entry, VectorCam has demonstrated an increase in data reporting efficiency, offering potential for national scale up, ultimately leading to better malaria control.

The technical performance of VectorCam and its feasibility have been established. However, widespread adoption of such technology requires an understanding of how to successfully implement, integrate, sustain, and scale the intervention in real-world health system contexts [4,26]. In Uganda and similar settings, implementation of novel technologies frequently fails or lacks sustainability, with common challenges stemming from technical (eg, infrastructure and expertise) and social (eg, culture, societal context, and human factors) dimensions [27,28]. This study uses an implementation science approach using the Consolidated Framework for Implementation Research (CFIR) and Expert Recommendations for Implementing Change (ERIC) strategies to assess and inform the VectorCam rollout, aiming to improve both uptake and sustainability of its implementation [29-32].

### Objectives

The main objective of this study is to evaluate how VectorCam can be sustainably integrated into Uganda’s national vector surveillance infrastructure across 12 districts, with a goal of informing future scale-up efforts across the country and in similar settings. The three aims of the study are to (1) identify contextual factors that enable the implementation of VectorCam in Uganda and evaluate the mechanisms through which they affect the implementation process; (2) assess the acceptability, feasibility, fidelity, and cost of VectorCam’s implementation model; and (3) evaluate the effectiveness of VectorCam in providing timely, accurate vector surveillance data and influencing malaria control decision-making.

## Methods

### Study Design Overview

This study uses a mixed methods, hybrid type III implementation-effectiveness design [33]. The primary aim is to evaluate implementation outcomes, including acceptability, feasibility, fidelity, and cost, while the secondary aim is to assess preliminary effectiveness outcomes, including technical accuracy of mosquito identification and operational use of VectorCam data in decision-making. Table 1 outlines these outcomes along with assessment methodology for each, demonstrating our multiple methods of data collection that will generate predefined key measures and are analyzed using both qualitative and quantitative approaches.

**Table 1:** Implementation and effectiveness outcomes, methods, and metrics by aim.

Outcome	Methods of data collection	Key measures	Planned analysis
Aim 1: assessment of contextual determinants	CFIR^a^-determinants evaluation survey with implementers; ERIC^b^ strategy matching tool Semistructured pre- and post-KIIs^c^ evaluating context with users^d^	CFIR barriers and facilitators by domain	Descriptive summaries of survey notes; quantitative summaries of Likert Scale ratings Thematic analysis of CFIR constructs; cross-district & cross-wave comparison matrices
Aim 2: acceptability, feasibility, fidelity (qualitative)	Semistructured pre- and post-KIIs evaluating implementation with users^d^ Midpoint IDIs^e^ with Master Trainers, VCOs^f^, and VHTs^g^ Quarterly stakeholder engagement meeting FRAME-IS^h^ documentation tool	Perceived usefulness, ease of use, workflow fit, perceived protocol adherence, sustained use, pain points, adaptation tracking	Thematic analysis; descriptive statistics; longitudinal summaries by district and rollout wave
Aim 2: fidelity (quantitative)	App usage and completeness metrics Field observation checklist and supervisory toolkit	Completeness of reporting; protocol adherence, district-level sustained use Protocol adherence rate	Proportions with 95% CIs; ordinal or binary mixed models where appropriate; district quarter trend plots
Aim 2: cost	Costing database, budgets, procurement and supervision records	Total and unit costs, budget impact, scale-up costs	Budget impact analysis modeling for 1-year, 3-year, and 5-year scenarios; ingredients-based costing; annualization of capital costs; one-way sensitivity analysis
Aim 3: technical effectiveness	Validation subset with VectorCam, routine method, and PCR^i^ (or equivalent)	Sensitivity, specificity, PPV^j^, NPV^k^, F1-score, agreement	Diagnostic accuracy analysis with 95% CIs; paired comparison when both methods are available; descriptive comparison of classification performance
Aim 3: operational effectiveness and data use	Quarterly NMED^l^ meeting attendance checklist KIIs to measure time allocation with users KIIs with district and national decision-makers	Use of data in planning; reporting timeliness Time allocation; task-shifting model metrics Data usage by decision-makers; impact on malaria control actions	Descriptive comparisons of time allocation metrics and data-use indicators across districts and rollout phases; mixed effects regression for time metrics; qualitative thematic analysis of data-use pathways

^a^CFIR: Consolidated Framework for Implementation Research.

^b^ERIC: Expert Recommendations for Implementing Change.

^c^KII: key informant interview.

^d^Pre and postinterviews are conducted 1-week after training and 3 months after implementation.

^e^IDI: in-depth interview.

^f^VCO: vector control officer.

^g^VHT: village health team.

^h^FRAME-IS: Framework for Reporting Adaptations and Modifications–Enhanced for Implementation Strategies.

^i^PCR: polymerase chain reaction.

^j^PPV: positive predictive value.

^k^NPV: negative predictive value.

^l^NMED: National Malaria Elimination Division.

**Figure 1:**
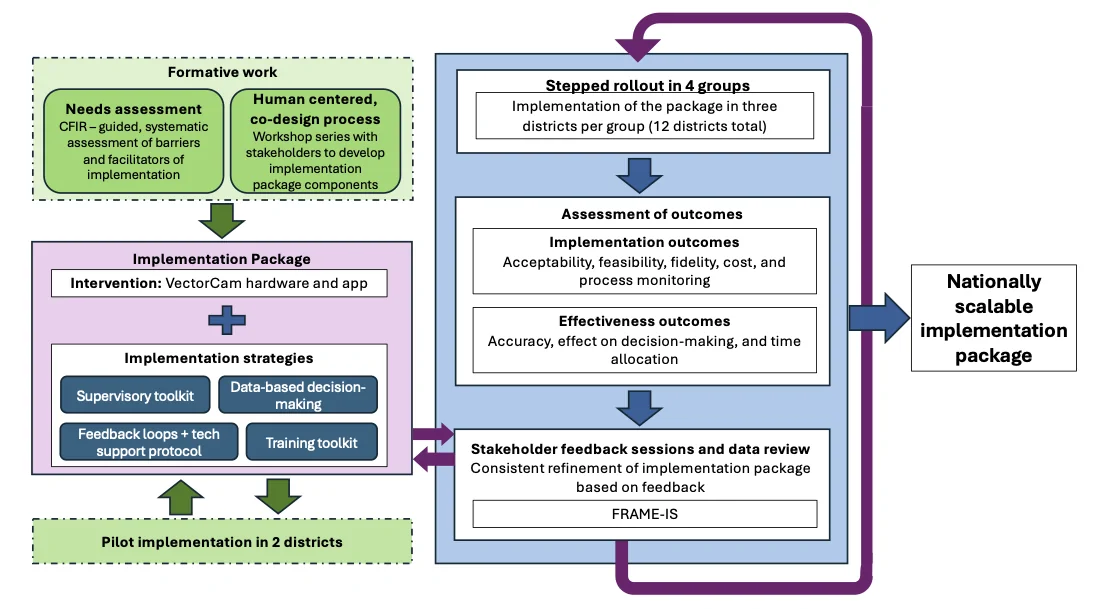
Conceptual overview of the hybrid type III implementation-effectiveness study design. CFIR: Consolidated Framework for Implementation Research; FRAME-IS: Framework for Reporting Adaptations and Modifications–Enhanced for Implementation Strategies.

The study includes a randomized rollout of VectorCam across a group of 3 districts every 3 months, supporting real-time learning and adaptations. The staggered rollout enables longitudinal assessment of implementation and effectiveness outcomes across districts and rollout waves. A midline review will synthesize this feedback to inform data-driven adaptations to the implementation package and technology design, subject to review and approval by Uganda’s MOH officials before rollout in the subsequent districts. Adaptations may include modifications to the intervention (eg, app updates) or to the implementation strategies (revised training toolkit), depending on identified needs. We apply a combination of complementary implementation science frameworks to guide design, evaluation, and adaptive refinement. Figure 1 illustrates the structure of this study, showing how implementation science theory will be used and the cyclic, stepped rollout that will allow us to systematically gather feedback and improve the intervention prior to each phase. This protocol aligns with the Standard Protocol Items: Recommendations for Interventional Trials (SPIRIT) guidelines, and the intervention description follows the Template for Intervention Description and Replication (TIDieR) checklist (Multimedia Appendices 1 and 2). A protocol timeline is provided in Multimedia Appendix 3.

### Study Setting and Participants

The MOH selected 14 districts in Uganda to receive VectorCam based on the following inclusion criteria: (1) Presence of diverse mosquito species relevant to malaria transmission; (2) workforce capacity - availability of trained VCOs and VHTs to participate in data collection; (3) district–level commitment to adopting digital vector surveillance tools; (4) willingness of local health authorities to support and sustain implementation; and (5) logistical feasibility to operate within the region (eg, security concerns, seasonal accessibility, and surveillance supply availability). A total of 2 districts, Koboko and Mayuge, were selected as pilot implementation districts to provide an opportunity to test and adapt the initial implementation package, methods of data collection, and key measures based on their high level of implementation readiness. Findings from the pilot will be reviewed jointly by the study team and the MOH to inform the refinement of research tools and implementation strategies. The selected districts represent a range of malaria transmission settings and implementation contexts while maintaining sufficient operational readiness to support the initial phase of national rollout. Although this study does not specifically target the most resource-constrained districts, a key objective is to identify the contextual determinants, implementation strategies, and support structures required for successful adoption of VectorCam. Findings from this study will inform future adaptation and scale-up efforts in settings with more limited surveillance infrastructure and workforce capacity.

The remaining 12 districts will implement the package in 4 sequential rollout phases, with 3 districts added every 3 months following completion of the pilot phase. This design enables evaluation of implementation processes across diverse contexts and over time, while allowing for iterative refinement of strategies between phases. Districts are assigned to rollout waves using restricted randomization at the district level via a computer-generated random sequence created by a study programmer prior to implementation. To ensure geographic balance, the allocation sequence will be constrained such that no rollout group includes more than one district from the same region. This approach was developed collaboratively by the study team and Uganda’s MOH to support evaluation across different districts while promoting equitable distribution of the intervention across regions during rollout. The rollout waves are intended to support implementation sequencing and adaptive learning rather than serve as comparison groups for causal inference. Multimedia Appendices 4 and 5 show information on the districts in each rollout group. To facilitate interpretation of our outcomes, contextual district characteristics, including malaria burden and workforce composition (eg number of VHTs and VCOs per district), will be documented during implementation activities and added descriptively to the table shown in Multimedia Appendix 5.

Key participants include VHTs, VCOs, master trainers (ie, district-level supervisors who are trained in the VectorCam system), District Health Officers, and staff from the National Malaria Elimination Division (NMED), representing multiple levels of the health system engaged in malaria surveillance and control.

### Intervention Description

The proposed task-shifting model for vector surveillance includes the VectorCam hardware and software (intervention) along with the strategies supporting its integration into Uganda’s routine vector surveillance program (implementation package). We used a human-centered design approach to developing the implementation package, with a particular focus on determining the details of the implementation strategies and materials. The implementation package was co-designed in consultation with national, district, and community-level stakeholders during a collaborative workshop held in August 2025. The participants included officials from the Ministry of Health, master (lead) VCOs, district VCOs, and members of the research team.

The intervention includes VectorCam imaging devices, smartphones preloaded with the VectorCam application, and charging accessories provided to each district and shown in Multimedia Appendix 6. The package includes 4 core implementation strategies: (1) a training toolkit and capacity building, (2) data-based decision making, (3) a supervisory toolkit and quality assurance, and (4) learning loops and procedures for ongoing technical support. These strategies are designed to address known barriers to implementation by improving user knowledge and skills, strengthening feedback and accountability systems, reinforcing perceived usefulness, and supporting integration into routine workflows. Because VectorCam is designed to support task shifting of surveillance activities to community-level personnel, the study will also evaluate the training intensity qualitatively and by number of retraining sessions completed, supervision requirements, technical support needs, and workflow adaptations necessary to maintain high-quality surveillance data. Figure 1 depicts the implementation package components conceptually.

Standardized training materials and the supervisory toolkit, including demonstrations, user manuals, inventory tracking procedures, and troubleshooting checklists, were codeveloped by the VectorCam implementation team, the researchers, and the MOH. Training will follow a cascade model in which Master Trainers from NMED will receive training on VectorCam operations, troubleshooting, supervision procedures, and supply management. These Master Trainers will then conduct district-level, hands-on training for VCOs and VHTs during the first week of rollout in the district. The training also includes pre- and posttraining knowledge assessments for VHTs that will identify any remaining gaps prior to VectorCam use in the district. Additionally, the supervisory toolkit includes a structured audit to assess protocol adherence, data quality, and equipment functionality. These toolkits are complemented by the data dashboards and monthly review meetings for regular feedback cadence. Technical support includes a tiered escalation pathway. First-line support is provided by VCOs, with second-line support from the VectorCam technical team for software issues or hardware failures. Procedures for reporting and resolving technical issues are documented using an “Implementation Issues Log” and reinforced during training. These strategies in combination are designed to support fidelity and acceptability of the intervention by improving consistency, knowledge, and quality of data collection.

After each implementation cycle, we will conduct structured stakeholder engagement meetings with district health teams, VCOs, and NMED representatives. This meeting cadence is built into the implementation package specifically to collect feedback from users, disseminate results, and share lessons learned. The Framework for Reporting Adaptations and Modifications–Enhanced for Implementation Strategies (FRAME-IS) will be used to document any significant changes to the implementation package, and these will be shared with stakeholders during the quarterly meeting [34]. Notes from these sessions will also help us identify facilitators and barriers to implementing and sustaining VectorCam, including factors related to resource availability, leadership support, and integration into routine workflows.

### Outcomes Assessment

Table 1 shows a breakdown of outcomes, methods for data collection, and evaluation metrics aligned with each aim. The table outlines how implementation and effectiveness will be assessed through mixed methods, ensuring that outcomes are aligned with the study’s overall evaluation framework. All surveys and interviews have been developed with feedback from the study’s in-country partners and will be administered with printed forms in either English or local languages as appropriate. The interviews will be administered by a researcher who is fluent in both English and the local language and trained to obtain consent before interviews.

**Figure 2:**
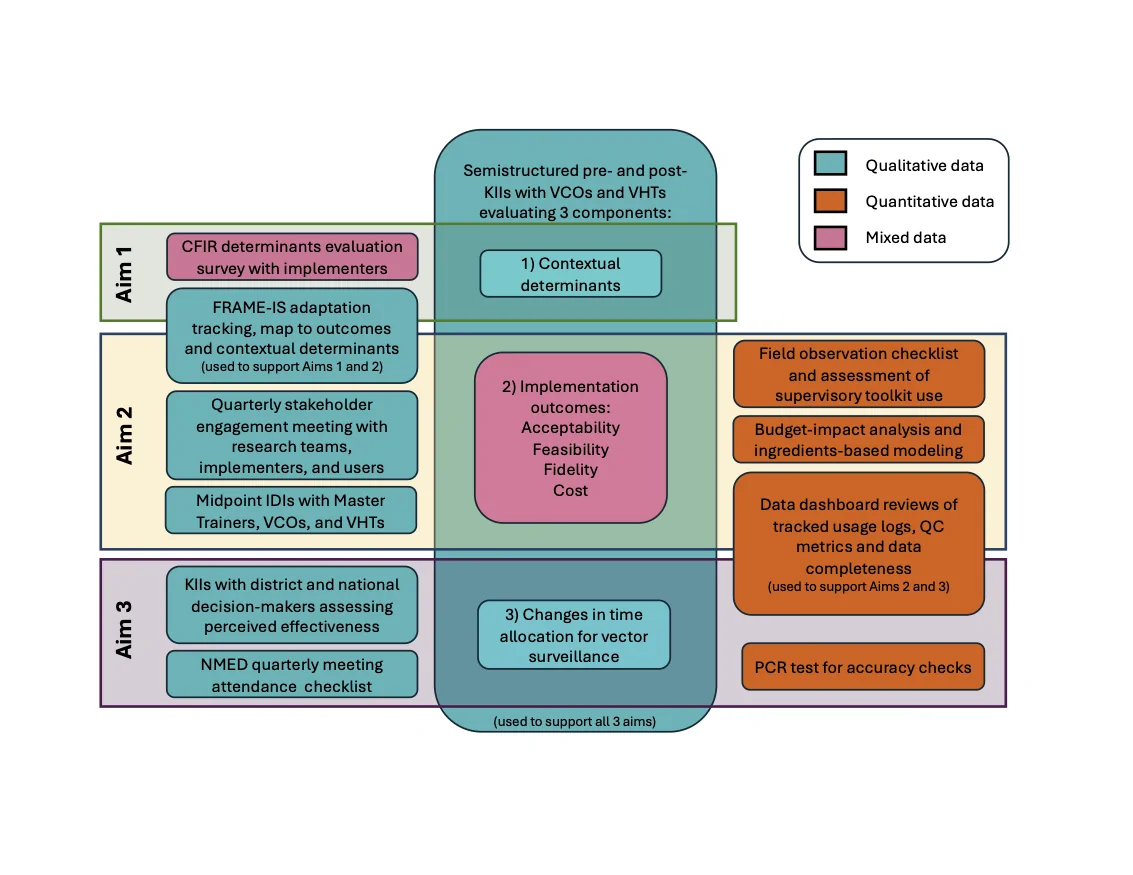
Overview of the mixed methods research tools used by aim. CFIR: Consolidated Framework for Implementation Research; IDI: in depth interview; KII: key informant interviews; NMED: National Malaria Elimination Division; PCR: polymerase chain reaction; QC: quality control; VCO: Vector Control Officers; VHT: village health teams.

Figure 2 illustrates all the methods of data collection used across the aims of this study, correlating with Table 1. An overview of each aim and a description of the methods used for data collection within them is detailed in the following sections.

### Aim 1: Evaluation of Contextual Determinants

We will assess the contextual determinants that influence implementation of the VectorCam platform by using an approach grounded in CFIR methodology and guided by established implementation mapping procedures [32]. This aim combines semistructured surveys and in-depth, pre- and post-key informant interviews (KIIs) to generate an understanding of the barriers and facilitators to successful implementation and evaluate the mechanisms through which they influence the implementation and effectiveness outcomes of interest.

Methods for data collection for Aim 1 include (1) CFIR-determinants evaluation survey with implementers, and (2) semistructured, pre- and post-KIIs to evaluate contextual characteristics with users. Firstly, for the CFIR-determinants evaluation surveys, we will administer a semistructured survey to select implementers: 4 MOH representatives and 6 VCOs or District Health Officials. This process will yield a total of 10 interviews conducted between months 4 and 6 of the staggered rollout. This survey will collect information on the context, probing for barriers and facilitators to implementation success. The survey instrument will primarily include Likert-scale questions to understand how the participant ranks the importance of specific determinants, supplemented by 5 open-ended questions to probe in-depth explanations for their choice of ranking. All questions are adapted from validated CFIR measurement tools to assess the 5 domains, including perceptions of intervention characteristics (eg, relative advantage and adaptability), inner setting (eg, organizational culture and implementation climate), outer setting (eg, external policies and community support), characteristics of individuals (eg, self-efficacy and knowledge), and process (eg, leadership engagement and planning).

Secondly, in each district, pre-KIIs will be conducted during the first week of intervention implementation (after training), and post-KIIs will then be administered 3 months later, focusing on the evaluation of contextual characteristics with users. Four VHTs and 2 VCOs will be purposely selected per district to ensure a balance of perspectives and varying levels of prior experience with entomological surveillance. The same participants will be contacted again for postimplementation interviews, enabling us to make paired comparisons of changes in attitudes and reported practices over time. Whenever possible, the same participants will be recontacted for follow-up interviews. If a participant is unavailable due to transfer, withdrawal, or other forms of attrition, a replacement participant occupying a similar implementation role will be purposively selected. Data collected through these sessions will inform iterative refinements to the implementation package and materials for subsequent adaptive cycles. By collecting rich qualitative data, the study team can examine what contextual determinants are most important during the initial phases of VectorCam rollout and, more precisely, how these factors are associated with changes in implementation outcomes.

### Aim 2: Acceptability, Feasibility, Fidelity, and Cost

The primary purpose of this aim is to ascertain the implementation outcomes of VectorCam use as defined by the Implementation-Outcome Framework [35]. Acceptability, fidelity, and feasibility of the implementation package will be assessed by using an integrated mixed methods approach that combines qualitative and quantitative data. For qualitative data, we will deploy pre- and postimplementation semistructured interviews, quarterly stakeholder engagement meeting notes, midpoint in-depth interviews with implementers and users, and FRAME-IS guided survey. Quantitative data collection tools for Aim 2 include data dashboard metrics, such as usage logs and percent completeness, and the field observation checklist. Additionally, the cost of VectorCam integration into Uganda’s vector surveillance system will be assessed with a combination of prospective costing methods, budget impact modeling, routine monitoring data, and structured feedback from district and national stakeholders. This aim will generate a comprehensive understanding of user experiences, protocol adherence, ease of use, fit with existing workflows, and evidence on the long-term financial feasibility of maintaining the implementation package at scale. Data from this aim can be used by Uganda’s MOH and its implementing partners to plan for a scalable and sustainable nationwide rollout.

Methods of data collection for Aim 2 include:

1. Semistructured pre- and post-KIIs to evaluate implementation outcomes with users
2. Field observation checklist to evaluate fidelity and supervisory toolkit
3. VectorCam app-system usage logs
4. Costing database, budgets, and procurement records
5. FRAME-IS documentation tool

The first data collection tool, pre- and post-KIIs, includes interview guides that are designed to be used at the same time points as the semistructured pre- and post-KII to evaluate context and administered to the same participants (ie, 4 VHTs and 2 VCOs) introduced in Aim 1 data collection methods. The purpose of this interview guide is to collect information about implementation outcomes. It will include structured questions for assessing satisfaction with the technology, perceived ease of use, alignment with existing workflows, confidence in the tool, and reflections on the frequency and consistency of use.

Each month, a VCO will use the second research tool and the field observation checklist to directly observe VHTs using the VectorCam system in the field to assess fidelity and the deployment of the supervisory toolkit. We will review these logs to verify the proportion of eligible VHTs trained in each cycle, equipment distribution, and posttraining support provided. The supervisory tool also contains a standardized field observation checklist and a set of structured questions designed to identify factors that may affect supervision, these questions will explore domains such as availability of logistical resources (eg, transport and devices), communication and coordination mechanisms, and context and barriers that will be used to assess how well VectorCam users are adhering to the protocol, including preparation and calibration of the device, correct specimen handling, adherence to identification procedures, and successful data upload to DHIS2. A VCO and a researcher will complete this checklist separately so that we will have 2 observations with which to assess district-level fidelity. Observers will rate protocol adherence as high, medium, or low and will provide narrative field notes describing deviations and potential contributing factors.

Quantitative data collected from VectorCam’s app will be used to assess percent of data completeness, and fidelity will be collected through system-generated usage logs exported monthly for analysis. Completeness of reporting and sustained use metrics will be summarized descriptively. Mixed-effects logistic or ordinal regression models may be used, where appropriate, to explore temporal patterns in completeness of reporting and implementation outcomes related to sustained use while accounting for clustering at the district level.

Throughout the study period, we will use a cost-tracking system to capture direct and indirect costs associated with VectorCam implementation in each district. Cost data will be collected prospectively and will include expenses related to procurement of hardware and consumables, training and supervision activities, technical support, and system maintenance. Financial records, procurement invoices, and project budgets will be reviewed to ensure comprehensive accounting of expenditures. Health economists on the study team will use these data to develop annual and multiyear budget projections, estimate total annual implementation costs, and calculate operational cost savings that result from replacement of manual surveillance methods. These models will generate 1-year, 3-year, and 5-year budget projections to inform national malaria control planning.

Lastly, to ensure transparent reporting of changes to the package over time, we will systematically document all modifications to the intervention and implementation strategies using the FRAME-IS [34]. Throughout implementation, project staff and implementing partners will record proposed or observed changes during routine monitoring and bi-weekly coordination calls. Each adaptation will be documented on a structured form aligned to FRAME-IS domains: (1) the nature of the modification (content, context, personnel, or delivery mode); (2) the level at which the change occurred (individual provider, facility, district, or system level); (3) whether the modification was planned or reactive; (4) the goal of the change, including improving feasibility, acceptability, fidelity, reach, or equity; (5) the decision-making process and actors involved; and (6) the extent to which the change was fidelity-consistent with core program components. We will then categorize adaptations into higher-level themes to enable comparison and identify patterns in how the implementation strategies evolved over time. This process will ensure transparent reporting of adaptations and allow linking of modifications to implementation outcomes and contextual drivers.

### Aim 3: Evaluation of Effectiveness

Effectiveness of the VectorCam intervention (as delivered with implementation strategies) in maintaining accuracy, improving time allocation metrics, and supporting operational use of surveillance data will be assessed with a combination of KIIs, quantitative analyses of system data logs, and qualitative interviews with national and district-level decision-makers. This aim is designed to generate preliminary evidence regarding VectorCam’s performance under ideal implementation conditions and whether the task-shifting model is associated with improvements in data quality and timelier, evidence-based decision-making relative to microscopy-based surveillance. Routine microscope-based entomological surveillance conducted by district entomology teams serves as the comparator condition, as it represents the standard of practice within Uganda’s national malaria surveillance system.

Methods of data collection for Aim 3 include:

1. Pre- and post-KIIs to evaluate time allocation with users
2. App-recorded data logging time spent per mosquito identification event
3. Molecular analysis comparison
4. KIIs with national and district-level decision-makers on operational effectiveness and data use
5. NMED quarterly meeting attendance and observational checklist

Pre- and postimplementation interviews with VHTs and VCOs will be used to assess the time required for mosquito identification and data submission. This tool is designed to be utilized at the same time point as the pre- and post-KIIs for data collection within Aims 1 and 2. Participants will be asked to describe the average time spent on identification tasks before and after the introduction of VectorCam, including steps related to specimen preparation, processing, and reporting. Questions on the KII guides differ for VHTs and VCOs in order to capture changes to workflow based on the task-shifting model.

Quantitative data on actual time spent per mosquito identification event will be extracted from VectorCam usage logs over the study period. Logs will include timestamps marking initiation of identification, confirmation of results, and data upload.

For the molecular analysis comparison, accuracy of mosquito species identification using VectorCam will be evaluated by comparing system-generated identifications to a gold-standard reference. Molecular identification will serve as the primary gold standard, using polymerase chain reaction (PCR) when species-specific reagents are available and sequencing when they are not. A random subset of 500 specimens collected during routine surveillance will be selected for molecular validation. This sample size was selected to provide sufficient precision for estimating identification accuracy under routine implementation conditions. Based on the 93% species identification accuracy observed in the previous RCT, a validation sample of 500 specimens yields an approximate 95% CI with a margin of error of +/- 2.2 percentage points around the overall accuracy estimate. These estimates will be used to characterize VectorCam performance descriptively where sample sizes are sufficient, recognizing that precision will vary by the number of specimens available for each species. These specimens will be analyzed independently by an entomologist blinded to the VectorCam results and PCR findings. Accuracy will be defined as the proportion of VectorCam identifications that match PCR results when available, or morphologic identification otherwise. In addition, model performance will be evaluated using standard classification metrics from an AI perspective. In addition to this primary accuracy measure, performance will also be evaluated using standard metrics, including precision, recall, and the *F*_1_-score, which together provide a balanced assessment of how well the model identifies mosquito species.

Next, we will explore VectorCam’s operational effectiveness and data use on malaria control decision-making by interviewing 8-10 MOH-NMED staff and District Health Officers. An interview guide was developed to facilitate the KIIs with national and district-level decision makers to collect qualitative information regarding operational effectiveness and VectorCam data use. The NMED groups that will be interviewed will either have routine interactions with the entomology database, responsibilities for improving the database, and/or responsibilities that involve using the database to make decisions about malaria control programs, including roles such as the Director of the National Malaria Program, Information Technology Senior Developer, lead entomologists, and coordinators of routine surveillance activities. Interviews will explore perceptions of the reliability and utility of VectorCam data, comparisons to previous surveillance approaches, and examples of how data informed vector control interventions or planning. Interviews will be conducted a minimum of 6 months after implementation in each district cohort to allow sufficient time for data review and use.

Lastly, a researcher from either Makerere University or Johns Hopkins University will attend NMED’s quarterly meetings and use the NMED quarterly meeting attendance and observational checklist to document how the VectorCam data are reviewed and used during routine decision-making processes. The checklist will capture whether VectorCam data were presented, discussed, or referenced in relation to surveillance planning, vector control activities, resource prioritization, or other operational decisions. Findings from these observations will be triangulated with key informant interviews to assess the perceived utility and operational use of VectorCam data.

### Data Management and Analysis

This study uses a convergent mixed methods analytic approach in which qualitative and quantitative data are collected and analyzed concurrently across all study aims. Data collection and analysis will occur iteratively across the staggered rollout cycles, allowing findings from earlier phases to inform subsequent data collection, refinement of tools, and interpretation of results. This approach is intended to capture the dynamic and context-dependent nature of implementation and to support adaptive learning throughout the study. Across all aims, data collected through multiple methods will be used to generate and validate key measures, and analyses will be conducted in alignment with the “Planned Analysis” column in Table 1.

All qualitative data, including semistructured key informant interviews, in-depth interviews, open-ended survey responses, and stakeholder engagement discussions, will be collected by trained research staff and transcribed verbatim when audio recording is permitted or expanded into detailed notes immediately following data collection. Research staff involved in observational data collection will receive standardized training on data collection procedures, use of observational tools, and documentation practices to promote consistency across observers and study sites. Data will be managed and analyzed using Atlas.TI. A single, unified codebook will be applied across all qualitative data sources to ensure consistency and enable comparison across stakeholder groups and time points. This approach is particularly important given that the same interview streams (ie, the pre and post-KIIs) contribute to multiple study aims and are intended to capture changes over time in determinants, implementation processes, and operational effectiveness.

Codebook development will follow a hybrid deductive and inductive approach (see Multimedia Appendix 7 for deductive codebook). Deductive codes will be derived from established implementation science frameworks, including the CFIR, as well as the Implementation Outcomes Framework (IOF) to capture constructs such as acceptability, feasibility, fidelity, and cost. After the first implementation rollout, the codebook will be iteratively refined to incorporate inductive codes that emerge from the data, including unanticipated barriers, contextual dynamics, adaptations, and potential district-specific codes. Coding will initially be conducted by at least 2 coders, with regular meetings to reconcile discrepancies, refine code definitions, and ensure consistent application of the coding framework. Prior to independent coding of the full dataset, coders will establish interrater reliability using a subset of transcripts. Interrater reliability will be assessed using percent agreement, with a minimum threshold of 0.75 required before proceeding to full coding. If agreement falls below this threshold, discrepancies will be reviewed jointly, code definitions will be refined, and additional training and calibration exercises will be conducted before reassessment. During coding, disagreements that cannot be resolved through discussion will be adjudicated by a senior member of the research team. Ongoing reflexive discussions will be used to examine assumptions and strengthen interpretive validity within the research teams. Framework analysis will be used to identify themes within and across stakeholder groups and over time, with particular attention to how contextual determinants influence implementation outcomes and how these relationships evolve across rollout cycles.

Quantitative data will be derived from structured surveys, field observation checklists, costing databases, and the data dashboard, which tracks key metrics such as data completeness. Descriptive analyses will be conducted to summarize key variables, including measures of data completeness, usage frequency, and accuracy. Implementation outcomes will be assessed using multiple complementary indicators and, where appropriate, proportions will be calculated with 95% CIs. Fidelity will be evaluated through both system-generated metrics, such as completeness of required data fields, and observational assessments of protocol adherence. Patterns of system use will be examined longitudinally to assess trends over time and variation across districts. Descriptive summaries will be generated by district and rollout wave, including district-quarter trend plots to visualize changes over time.

Cost analysis will be conducted from the program perspective, with scenario analyses for broader health system scale-up. An ingredients-based costing approach will be used to estimate startup and recurrent costs, including hardware, consumables, training, supervision, maintenance, connectivity, and technical support. Capital items will be annualized over their useful life using a standard discount rate. Unit costs will be reported per district covered, per user trained, and per identification event completed. Budget impact models will be developed to project costs under different scale-up scenarios, incorporating both observed expenditures and anticipated changes in resource requirements. Sensitivity analyses will test uncertainty in key assumptions.

Effectiveness outcomes will be assessed using both quantitative and qualitative data. Time allocation will be evaluated through both self-reported data (collected via the pre- and post- KIIs) and system-recorded measures of time spent on surveillance activities. To evaluate the task-shifting model that VectorCam used, time allocation comparisons will be made across pre- and postimplementation periods. Accuracy of mosquito identification will be assessed by comparing VectorCam outputs to the gold-standard molecular identification (PCR or sequencing for species for which PCR reagents are not available). Qualitative data from interviews with decision-makers will be analyzed thematically to assess the perceived utility of surveillance data for decision-making using the same codebook as other qualitative data collection tools.

Integration of qualitative and quantitative data will occur throughout the analytic process to enable triangulation and strengthen the validity of findings. This will occur through joint displays, triangulation matrices, and iterative comparison of quantitative and qualitative findings. Quantitative results will be interpreted alongside qualitative insights to assess convergence and to provide context for observed patterns. Qualitative data will also be used to explain unexpected quantitative findings and to identify mechanisms underlying observed relationships. Integration will be further supported using an Implementation Research Logic Model, which will link contextual determinants, implementation strategies, and outcomes across data sources.

Documentation of adaptations using FRAME-IS will be analyzed alongside implementation and effectiveness metrics to examine whether changes to the implementation approach are associated with improvements in the implementation outcomes we are analyzing. Comparisons across stakeholder groups will also be conducted to assess alignment between frontline experiences, implementation processes, and decision-making.

Through this integrated analytic approach, the study will generate a comprehensive understanding of how VectorCam is implemented within routine surveillance systems, how implementation processes influence outcomes, and how improvements in data quality and timeliness translate into actionable use for malaria control.

### Sample Size and Power

Across all aims, sample size determination is guided by the descriptive and implementation-focused nature of the study rather than formal hypothesis testing. In each of the 12 districts, 4 VHTs and 2 VCOs will participate in pre- and postimplementation qualitative assessments to collect data across all 3 aims. These KIIs will yield a total of 72 interviews at baseline and 72 at follow-up. The sample size was selected to ensure representation of the primary implementation actors across all participating districts while remaining feasible within a national implementation study. Participants will be purposively selected to capture perspectives across diverse implementation contexts, and the anticipated number of interviews is expected to provide sufficient breadth and depth to identify recurring themes related to implementation processes and outcomes. In addition, approximately 10 KIIs will be conducted with district-level and national decision-makers to assess effectiveness for Aim 3. We again expect this number will be sufficient to achieve thematic saturation for our (preliminary) effectiveness outcomes of interest. Quantitative system data logs will include all identification records generated by trained VHTs during the study period, and for accuracy validation, at least 500 specimens will be randomly selected and assessed across all districts. Because the study primarily employs descriptive rather than hypothesis-driven testing, no formal power calculations were conducted. The overall sample is expected to be sufficient to generate estimates of implementation outcomes and detect meaningful differences in time efficiency, identification accuracy, and data usage.

### Ethical Considerations

This study has received ethical approval from the Johns Hopkins Medicine Institutional Review Board (protocol number IRB00505343) and, in Uganda, from the AIDS Support Organization (TASO) Research Ethics Committee (TASO-2025-879) and the Makerere University School of Public Health Research Review Committee. The VectorCam device was reviewed and determined to be a nonsignificant risk (NSR) device meeting criteria for an abbreviated investigational device exemption. No significant harms are anticipated due to the minimal-risk nature of the study.

This study is embedded within ongoing Ministry of Health routine entomological surveillance activities. For components involving human participants (eg, interviews, surveys, and observational assessments), written informed consent is obtained prior to participation. Consent forms are available in English and in relevant local languages (including Lusoga, Ngakarimojong, Luo, and Lugbara), and trained research staff conduct consent discussions in the participant’s preferred language, using certified translations. Participants who complete interviews or surveys will receive 20,000 Ugandan Shillings (UGX) as compensation for their time. The compensation amount was reviewed and approved by the relevant ethics committees and is not considered coercive.

At the start of the consent process, participants will be asked to indicate what language they are comfortable with, and the appropriate consent forms will be selected. All sections of the consent document will be read verbatim in the selected language and explained to ensure comprehension. All participants will be informed that participation in the study is entirely voluntary and that they may withdraw from the study at any time. Signed consent forms are retained in secure study files in accordance with institutional and national regulatory requirements.

All identifiable data will be stored on secure, password-protected servers at Johns Hopkins University and Makerere University, accessible only to authorized study personnel. Deidentified data will be used for analysis, and no identifying information will be shared or published.

## Results

In collaboration with Uganda’s Ministry of Health, the study team identified 22 districts that met the inclusion criteria, from which 14 districts were selected to receive the VectorCam intervention in September 2025. The pilot phase was conducted from December 2025 to January 2026, during which research tools were tested during rollout in two districts. Stakeholder workshops in the pilot phase were used to refine the implementation package and research instruments. Ethics approval was granted in January 2026. Data collection for the first rollout group began in February 2026, initiating a year-long data collection period. As of March 2026, the second rollout group of 3 districts is scheduled to begin data collection in June 2026. Data collection and subsequent analysis will be concurrent in districts where the intervention has been deployed and are expected to continue through March 2027.

## Discussion

### Anticipated Findings

This protocol represents one of the first hybrid type III studies to evaluate how an AI-enabled surveillance tool can be scaled and sustained within a national vector control program in low- and middle-income countries (LMICs). Building on strong feasibility evidence from VectorCam’s prior RCT, we will assess how contextual determinants influence uptake, identify and refine implementation strategies, and generate robust evidence on both implementation outcomes and effectiveness across diverse malaria-endemic districts in Uganda. The study is designed to contribute to the evidence base on implementation of AI-enabled digital health innovations in routine public health systems of LMICs.

Beyond evaluating digital surveillance technology, this study examines a task-shifting model in which community-level personnel conduct activities traditionally performed by trained entomologists. Understanding the contextual determinants, implementation strategies, training requirements, and supervision structures associated with successful implementation will be critical for future expansion of surveillance activities into districts with more limited workforce capacity and infrastructure. Although the current rollout focuses on districts selected for implementation readiness, findings from this study are intended to inform future deployment in underserved settings where entomological surveillance remains limited or unavailable.

Several features strengthen the rigor and relevance of this study. First, our implementation strategy development is grounded in the CFIR and the ERIC taxonomy, ensuring a theory-driven and context-sensitive approach to scale-up. Use of the FRAME-IS allows systematic documentation of adaptations to the implementation model, enabling us to address the growing demand for transparency and reproducibility in implementation research. Additionally, we integrate learning processes across all phases of the study to support iterative adaptation and stakeholder co-ownership, which bolsters the probability of long-term sustainability.

### Challenges and Limitations

The reliance on system usage logs and structured observations as proxies for implementation quality may underestimate informal use or undocumented fidelity. However, triangulation with qualitative interviews, cost tracking, and FRAME-IS documentation is expected to provide a more complete picture.

Another limitation is that data from VHTs and VCOs will be collected through self-report and field observations, both of which may be influenced by social desirability bias or observer effects. To mitigate this limitation, researchers will undergo structured training, and all the survey tools will be codeveloped, pilot tested, or both. Additionally, this implementation represents the inaugural national rollout of VectorCam into Uganda's routine vector surveillance program, no prior equivalent program exists against which a concurrent or historical control comparison could be meaningfully constructed. Thus, effectiveness outcomes should be interpreted as preliminary and descriptive rather than causal.

Although VectorCam has demonstrated strong technical performance in prior trials, scale-up may introduce new technological or bandwidth challenges not previously encountered. The monitoring, evaluation, and learning framework and staggered implementation schedule will help mitigate this risk through continuous monitoring and rapid-cycle problem solving. Finally, although the budget impact analysis will model cost scenarios at 1- and 5-year horizons, longer-term sustainability will also depend on shifts in MOH budget allocations and digital infrastructure, which are factors outside the scope of this study but relevant for future planning.

### Conclusions

This study represents an important contribution to implementation science, digital public health, and malaria vector control, as we aim to evaluate the implementation and effectiveness of a co-designed, AI-enabled platform integrated within Uganda’s national malaria surveillance system. Through the examination of contextual determinants, implementation strategies and outcomes, and preliminary effectiveness outcomes, this study seeks to generate evidence on the feasibility of task-shifting routine entomological surveillance activities to community-level personnel. The insights generated from utilizing this protocol for Uganda’s VectorCam rollout can inform the scale-up of similar surveillance technologies across LMICs and help accelerate progress toward malaria elimination.

## Appendix

Multimedia Appendix 1 SPIRIT checklist 2025.

Multimedia Appendix 2 TIDieR checklist for Protocols.

Multimedia Appendix 3 Protocol timeline describing research and implementation activities and the staggered rollout design.

Multimedia Appendix 4 Map with Ugandan districts and rollout groups depicted.

Multimedia Appendix 5 This table indicates the districts receiving the VectorCam implementation package and sorts them by rollout group.

Multimedia Appendix 6 Depiction of the novel VectorCam intervention device and smart phone application.

Multimedia Appendix 7 Preliminary deductive codebook used for the protocol's qualitative analyses.

## Abbreviations

AED: Aedes spp.
AF: Anopheles funestus s.l.
AG: Anopheles gambiae s.l.
AO: Anopheles Other
CDC-LTC: Centers for Disease Control light trap catches
CFIR: Consolidated Framework for Implementation Research
CNN: convolutional neural network
CU: Culex spp.
DHIS2: District Health Information System 2
ERIC: Expert Recommendations for Implementing Change
FRAME-IS: Framework for Reporting Adaptations and Modifications–Enhanced for Implementation Strategies
HLC: human-landing catch
IOF: Implementation Outcomes Framework
KII: key informant interview
LMIC: low- and middle-income country
MAN: Mansonia spp.
MOH: Ministry of Health
NMED: National Malaria Elimination Division
NSR: nonsignificant risk
PCR: polymerase chain reaction
PSCs: pyrethrum spray catches
RCT: randomized controlled trial
SPIRIT: Standard Protocol Items: Recommendations for Interventional Trials
TASO: AIDS Support Organization
TIDieR: Template for Intervention Description and Replication
VCOs: vector control officers
VHT: village health team

## Data Availability

No datasets have yet been generated, as this manuscript reports a study protocol. All research-related datasets, study instruments, implementation materials, codebooks, data dictionaries, and analytic code used to generate published findings will be made publicly available through an open-access repository (eg, OSF) upon publication of primary study results. Where permitted by ethical, regulatory, and Ministry of Health requirements, deidentified datasets or aggregate data supporting published findings may be made available through a controlled-access request process.
