# Supplementary material for: Integration of VectorCam, an AI-Enabled Mosquito Surveillance Tool, Into Uganda’s National Malaria Elimination Program: Protocol for a Mixed Methods Hybrid Type III Implementation Study
Source: JMIR Public Health Surveill. 2026 Sep 10;12:e99717. doi: 10.2196/99717 (PMC13613040; doi:10.2196/99717)
Supplement: Supplementary file 2 [file publichealth_v12i1e99717_app2.pdf]

TIDieR

Template for Intervention Description and Replication

The TIDieR (Template for Intervention Description and Replication) Checklist*:

Information to include when describing an intervention and the location of the information

Item number	Item	Where located ** Primary paper (page or appendix number)	Other † (details)
	**BRIEF NAME**		
**1.**	Provide the name or a phrase that describes the intervention.	10, 11	VectorCam™ digital mosquito identification and surveillance implementation package.
	**WHY**		
**2.**	Describe any rationale, theory, or goal of the elements essential to the intervention.	5 - 8	See Background section of protocol.
	**WHAT**		
**3.**	Materials: Describe any physical or informational materials used in the intervention, including those provided to participants or used in intervention delivery or in training of intervention providers. Provide information on where the materials can be accessed (e.g. online appendix, URL).	10, 11	Materials include: (1) VectorCam™ hardware device for AI-enabled mosquito image capture and a smart phone; (2) associated software and cloud-based analytics platform; (3) implementation blueprint; (4) training manuals and slide decks; (5) supervisory monitoring tools, which include a list of consumables necessary to have in stock at each district location (e.g. Eppendorf tubes, labels, power banks, masking tape, PSC sheets, Ethanol, petri dishes, PPE), ; (6) data dashboards for monitoring fidelity and outcomes; (7) Assessment tools used to collect qualitative and quantitative data in this protocol. Supplementary materials, including the assessment tools, are provided in Additional Files of this manuscript.

**4.**	Procedures: Describe each of the procedures, activities, and/or processes used in the intervention, including any enabling or support activities.	8-21	Procedures include: (1) baseline assessment; (2) co-design workshop with Ministry of Health, Makarere University, and district stakeholders; (3) training of district-level personnel; (4) installation and deployment of VectorCam™ devices on a staggered, district-level rollout; (6) routine mosquito collection and AI-based identification; (7) supervisory monitoring and process evaluation, including midpoint review of data and interviews with users and trainers; (8) collection of implementation outcome and effectiveness data; (9) iterative adaptation informed by FRAME-IS and monitoring, evaluation, and learning (MEL) processes; and 10) cost tracking and budget modelling for informing future scale-up
	**WHO PROVIDED**		
**5.**	For each category of intervention provider (e.g. psychologist, nursing assistant), describe their expertise, background and any specific training given.	9-12	The intervention will be delivered by district-level entomological surveillance officers designated as Master Trainers within each participating district. These individuals are existing Ministry of Health personnel responsible for routine mosquito collection and surveillance activities and typically have training in entomology, vector control, or environmental health. Initial training on VectorCam™ operation, data reporting, and implementation procedures will be conducted by the Johns Hopkins University research team in collaboration with Makerere University researchers. Master Trainers will subsequently train additional district-level staff and community health workers involved in mosquito collection and identification. Ongoing supervisory support and technical assistance will be provided by the research team and Ministry of Health collaborators throughout the rollout period.

	**HOW**		
**6.**	Describe the modes of delivery (e.g. face-to-face or by some other mechanism, such as internet or telephone) of the intervention and whether it was provided individually or in a group.	9-12	The intervention is delivered through in-person training sessions, on-site device deployment, and ongoing supervisory support. VectorCam™ use occurs individually by surveillance personnel during routine mosquito collection activities. Monitoring and feedback occur through digital dashboards and scheduled review meetings.
	**WHERE**		
**7.**	Describe the type(s) of location(s) where the intervention occurred, including any necessary infrastructure or relevant features.	9, 10	The intervention will be implemented in 14 malaria-endemic districts in Uganda selected by the Ministry of Health. Activities occur within existing district-level entomological surveillance infrastructure, including health facilities and field collection sites. Necessary infrastructure includes electricity for device charging, internet connectivity for data synchronization (where available), and existing surveillance workflows.
	**WHEN and HOW MUCH**		
**8.**	Describe the number of times the intervention was delivered and over what period of time including the number of sessions, their schedule, and their duration, intensity or dose.	11-20	Following a pilot in two districts, the remaining 12 districts will be enrolled in four sequential rollout phases, with three districts added every three months. VectorCam™ will be integrated into routine surveillance activities and used continuously throughout the study period. Training occurs prior to district rollout, with ongoing supervisory monitoring during implementation.
	**TAILORING**		
**9.**	If the intervention was planned to be personalised, titrated or adapted, then describe what, why, when, and how.	13-15	The intervention includes planned contextual tailoring based on CFIR-identified determinants. Implementation strategies may be adapted across districts to improve contextual fit. Adaptations will be systematically documented using the FRAME-

			IS framework and informed by monitoring, evaluation, and stakeholder feedback processes.
	**MODIFICATIONS**		
**10.‡**	If the intervention was modified during the course of the study, describe the changes (what, why, when, and how).	N/A	This is a protocol paper, and the study has not yet taken place. However, all modifications to the intervention will be documented (prospectively, wherever possible) using FRAME-IS and disseminated at the quarterly stakeholder engagement meetings that are included on the proposed study timeline.
	**HOW WELL**		
**11.**	Planned: If intervention adherence or fidelity was assessed, describe how and by whom, and if any strategies were used to maintain or improve fidelity, describe them.	N/A	This is a protocol paper, and the study has not yet taken place. Fidelity will be assessed by collecting data from usage logs, the supervisory tool and field observation checklist as described in Table 1. The supervisory tool is completed by a district-level supervisor (i.e. not a researcher), but during this study, a researcher will use the field observation checklist to assess the fidelity of the supervisory tool itself. As part of the process monitoring plan, contextual assessment will be conducted at the midpoint of the study by researchers at Makerere University using CFIR-informed tools. This assessment will then be used to update, modify, and/or select additional implementation strategies that are deemed critical to adherence and fidelity.
**12.‡**	Actual: If intervention adherence or fidelity was assessed, describe the extent to which the intervention was delivered as planned.		This is a protocol paper, and the study has not yet taken place.

** **Authors** - use N/A if an item is not applicable for the intervention being described. **Reviewers** – use '?' if information about the element is not reported/not sufficiently reported.

† If the information is not provided in the primary paper, give details of where this information is available. This may include locations such as a published protocol or other published papers (provide citation details) or a website (provide the URL).

‡ If completing the TIDieR checklist for a protocol, these items are not relevant to the protocol and cannot be described until the study is complete.

* We strongly recommend using this checklist in conjunction with the TIDieR guide (see *BMJ* 2014;348:g1687) which contains an explanation and elaboration for each item.

* The focus of TIDieR is on reporting details of the intervention elements (and where relevant, comparison elements) of a study. Other elements and methodological features of studies are covered by other reporting statements and checklists and have not been duplicated as part of the TIDieR checklist. When a **randomised trial** is being reported, the TIDieR checklist should be used in conjunction with the CONSORT statement (see www.consort-statement.org) as an extension of **Item 5 of the CONSORT 2010 Statement.** When a **clinical trial protocol** is being reported, the TIDieR checklist should be used in conjunction with the SPIRIT statement as an extension of **Item 11 of the SPIRIT 2013 Statement** (see www.spirit-statement.org). For alternate study designs, TIDieR can be used in conjunction with the appropriate checklist for that study design (see www.equator-network.org).
