# Supplementary material for: Integration of VectorCam, an AI-Enabled Mosquito Surveillance Tool, Into Uganda’s National Malaria Elimination Program: Protocol for a Mixed Methods Hybrid Type III Implementation Study
Source: JMIR Public Health Surveill. 2026 Sep 10;12:e99717. doi: 10.2196/99717 (PMC13613040; doi:10.2196/99717)
Supplement: Supplementary file 3 [file publichealth_v12i1e99717_app3.pdf]

Table: Protocol Timeline

			Month																													
	Activity	Pre-Implementation	0	1	2	3	4	5	6	7	8	9	10	11	12	13	14	15	16	17	18	19	20	21	22	23	24	25	26	27	28	29
Pre-Rollout	Pre-Visit to the district (District Checklist)	O		O																												
	CFIR Surveys & KIIs	O																														
	Co-Design Workshop	O							X																							
	ERIC Strategy to CFIR Constructs Matching Tool	O																														
Pilot Districts	CFIR Surveys & KIIs		O	O																												
	District visit and supplies checklist, baseline data		O																													
	Training & Rollout		O																													
	Pre-KII Evaluating Context, Implementation and Time Allocation			O																												
	Field Observation Checklist for District-Level Fidelity			O		X			X					X						X												
	Monthly Supervisory Tool Deployed (during first 5 days of the month)			O	O	X	X	X	X	X	X	X	X	X	X	X	X	X	X	X	X	X	X	X	X	X	X	X	X	X	X	X
	Usage Logs Extracted			O	O	X	X	X	X	X	X	X	X	X	X	X	X	X	X	X	X	X	X	X	X	X	X	X	X	X	X	X
	Cost Tracking			O	O	X	X	X	X	X	X	X	X	X	X	X	X	X	X	X	X	X	X	X	X	X	X	X	X	X	X	X
	Post-KII Evaluating Context, Implementation and Time Allocation					X								X																		
	Quarterly Meeting Attendance Tracker & Checklist Evaluating MOH Fidelity					X			X		X			X			X			X												
	Midpoint Process Monitoring KII with VHTs and VCOs								X					X																		
	FRAME-IS Documentation								X					X																		
	Stakeholder Engagement Meeting to review data and changes needed						X			X				X																		
Group 1 of QTY (3) Districts	District Request of supplies, baseline data																															
	District visit and supplies checklist					X																										
	Training & Rollout					X																										
	Pre-KII Evaluating Context, Implementation and Time Allocation						X																									
	Field Observation Checklist for District-Level Fidelity						X		X		X						X						X									
	Monthly Supervisory Tool Deployed (during first 5 days of the month)						X	X	X	X	X	X	X	X	X	X	X	X	X	X	X	X	X	X	X	X	X	X	X	X	X	X
	Usage Logs Extracted						X	X	X	X	X	X	X	X	X	X	X	X	X	X	X	X	X	X	X	X	X	X	X	X	X	X
	Cost Tracking						X	X	X	X	X	X	X	X	X	X	X	X	X	X	X	X	X	X	X	X	X	X	X	X	X	X
	Post-KII Evaluating Context, Implementation and Time Allocation								X								X															
	Quarterly Meeting Attendance Tracker & Checklist Evaluating MOH Fidelity								X		X			X			X			X			X									
	Midpoint Process Monitoring KII with VHTs and VCOs										X						X															
	FRAME-IS Documentation										X						X															
	Stakeholder Engagement Meeting to review data and changes needed								X		X						X															
Group 2 of QTY (3) Districts	Gather baseline data, supplies checklist								X																							
	Training & Rollout								X																							
	Pre-KII Evaluating Context, Implementation and Time Allocation									X																						
	Field Observation Checklist for District-Level Fidelity									X	X			X						X						X						
	Monthly Supervisory Tool Deployed									X	X	X	X	X	X	X	X	X	X	X	X	X	X	X	X	X	X	X	X	X	X	X
	Usage Logs Extracted									X	X	X	X	X	X	X	X	X	X	X	X	X	X	X	X	X	X	X	X	X	X	X
	Cost Tracking									X	X	X	X	X	X	X	X	X	X	X	X	X	X	X	X	X	X	X	X	X	X	X
	Post-KII Evaluating Context, Implementation and Time Allocation										X									X												
	Quarterly Meeting Attendance Tracker & Checklist Evaluating MOH Fidelity										X			X			X			X			X			X						
	Midpoint Process Monitoring KII with VHTs and VCOs													X						X												
	FRAME-IS Documentation													X						X												
	Stakeholder Engagement Meeting to review data and changes needed										X			X						X												
Group 3 of QTY (3) Districts	Gather baseline data, supplies checklist										X																					
	Training & Rollout										X																					
	Pre-KII Evaluating Context, Implementation and Time Allocation											X																				
	Field Observation Checklist for District-Level Fidelity											X		X			X						X						X			
	Monthly Supervisory Tool Deployed											X	X	X	X	X	X	X	X	X	X	X	X	X	X	X	X	X	X	X	X	X
	Usage Logs Extracted											X	X	X	X	X	X	X	X	X	X	X	X	X	X	X	X	X	X	X	X	X
	Cost Tracking											X	X	X	X	X	X	X	X	X	X	X	X	X	X	X	X	X	X	X	X	X
	Post-KII Evaluating Context, Implementation and Time Allocation													X									X									
	Quarterly Meeting Attendance Tracker & Checklist Evaluating MOH Fidelity													X			X			X			X			X			X			
	Midpoint Process Monitoring KII with VHTs and VCOs																X						X									
	FRAME-IS Documentation																X						X									
	Stakeholder Engagement Meeting to review data and changes needed													X			X						X									
Group 4 of QTY (3) Districts	Gather baseline data, supplies checklist													X																		
	Training & Rollout													X																		
	Pre-KII Evaluating Context, Implementation and Time Allocation														X																	
	Field Observation Checklist for District-Level Fidelity														X		X			X						X						X
	Monthly Supervisory Tool Deployed														X	X	X	X	X	X	X	X	X	X	X	X	X	X	X	X	X	X
	Usage Logs Extracted														X	X	X	X	X	X	X	X	X	X	X	X	X	X	X	X	X	X
	Cost Tracking														X	X	X	X	X	X	X	X	X	X	X	X	X	X	X	X	X	X
	Post-KII Evaluating Context, Implementation and Time Allocation																X									X						
	Quarterly Meeting Attendance Tracker & Checklist Evaluating MOH Fidelity																X			X			X			X			X			X
	Midpoint Process Monitoring KII with VHTs and VCOs																			X						X						
	FRAME-IS Documentation																			X						X						
	Stakeholder Engagement Meeting to review data and changes needed																X			X						X						
	KII Interview Guide to Evaluate Perceived Effectiveness								X					X						X												

O	Tools Piloted
X	Data Collected
(yellow)	In country
(green)	Online
(purple)	Master Trainers
(blue)	Other
