# Supplementary material for: Integration of VectorCam, an AI-Enabled Mosquito Surveillance Tool, Into Uganda’s National Malaria Elimination Program: Protocol for a Mixed Methods Hybrid Type III Implementation Study
Source: JMIR Public Health Surveill. 2026 Sep 10;12:e99717. doi: 10.2196/99717 (PMC13613040; doi:10.2196/99717)

VectorCam - Uganda National Rollout Districts

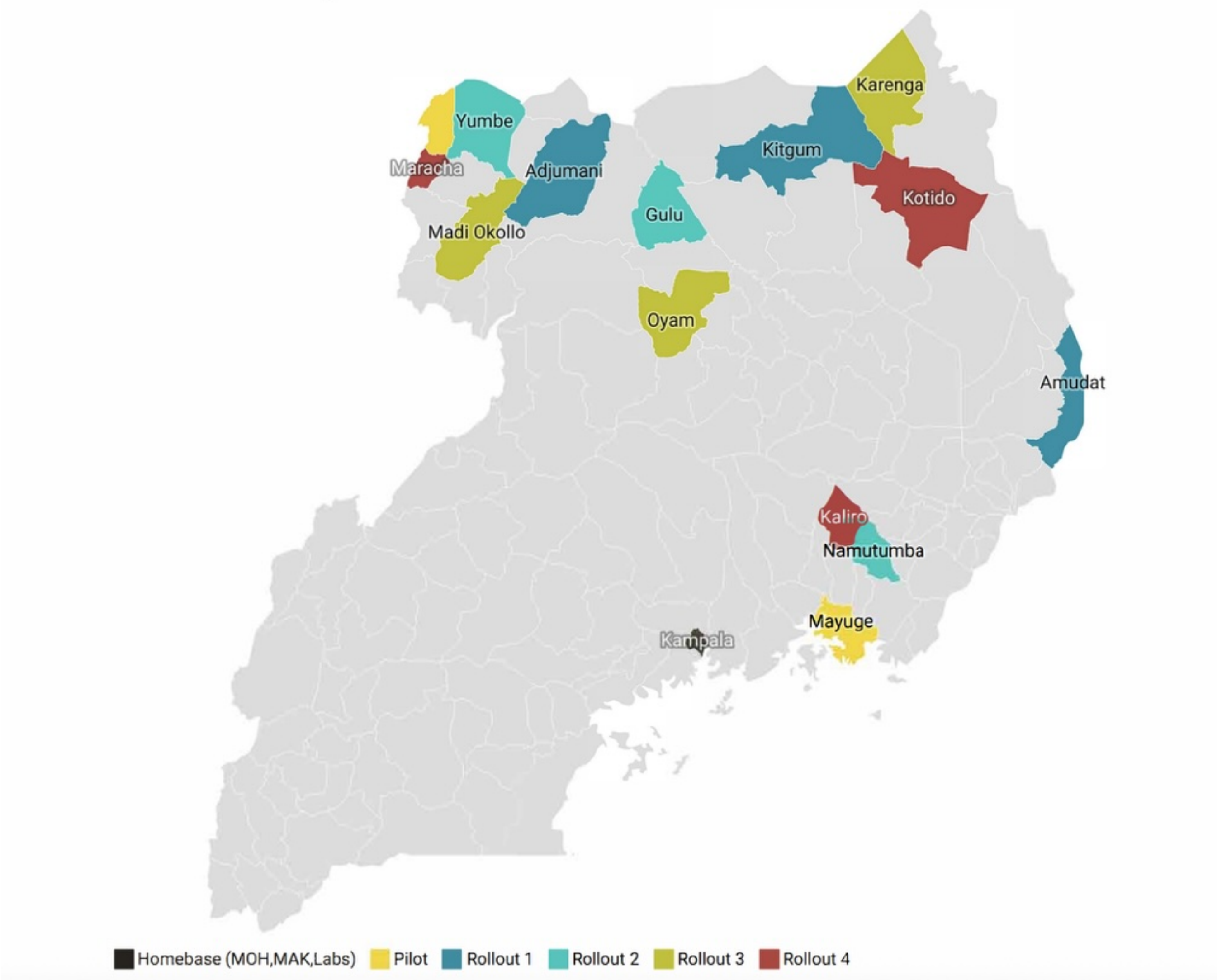

Supplement: Supplementary file 4 [file publichealth_v12i1e99717_app4.pdf]
