# Supplementary material for: Integration of VectorCam, an AI-Enabled Mosquito Surveillance Tool, Into Uganda’s National Malaria Elimination Program: Protocol for a Mixed Methods Hybrid Type III Implementation Study
Source: JMIR Public Health Surveill. 2026 Sep 10;12:e99717. doi: 10.2196/99717 (PMC13613040; doi:10.2196/99717)
Supplement: Supplementary file 5 [file publichealth_v12i1e99717_app5.pdf]

Table: Districts by Rollout Group and Baseline Characteristics

<table>
<tr><th colspan="3"></th><th colspan="4">Infrastructure Level</th><th colspan="4">Workforce Capacity</th><th>Malaria Burden</th></tr>
<tr><th>Rollout #</th><th>Region</th><th>District</th><th>Wifi (yes/No)</th><th>Electricity (Yes/No)</th><th>Consistent access to supplies or supply storage (Yes/No)</th><th>Access to vehicles or motorbikes (Yes/No)</th><th>Number of VHTs</th><th>Number of CHEWs</th><th>Number of VCOs</th><th>Number of health assistants</th><th>Rated as high, medium or low</th></tr>
<tr><td rowspan="2">Pilot</td><td>Busoga</td><td>Mayuge</td><td rowspan="15" colspan="9">Baseline Data Collection TBD</td></tr>
<tr><td>West Nile</td><td>Koboko</td></tr>
<tr><td rowspan="3">1</td><td>Acholi</td><td>Kitgum</td></tr>
<tr><td>Karamoja</td><td>Amudat</td></tr>
<tr><td>West Nile</td><td>Adjumani</td></tr>
<tr><td rowspan="3">2</td><td>Acholi</td><td>Gulu</td></tr>
<tr><td>Busoga</td><td>Namutumba</td></tr>
<tr><td>West Nile</td><td>Yumbe</td></tr>
<tr><td rowspan="3">3</td><td>Acholi</td><td>Oyam</td></tr>
<tr><td>Karamoja</td><td>Karenga</td></tr>
<tr><td>West Nile</td><td>Madi-Okollo</td></tr>
<tr><td rowspan="3">4</td><td>Busoga</td><td>Kaliro</td></tr>
<tr><td>Karamoja</td><td>Kotido</td></tr>
<tr><td>West Nile</td><td>Maracha</td></tr>
</table>
